# Focal sharp waves are a specific early-stage marker of the MM2-cortical form of sporadic Creutzfeldt-Jakob disease

**DOI:** 10.1080/19336896.2020.1803516

**Published:** 2020-08-13

**Authors:** Taiki Matsubayashi, Miho Akaza, Yuichi Hayashi, Tsuyoshi Hamaguchi, Masahito Yamada, Takayoshi Shimohata, Takanori Yokota, Nobuo Sanjo

**Affiliations:** a Department of Neurology and Neurological Science, Tokyo Medical and Dental University Graduate School of Medical and Dental Sciences, Tokyo, Japan; b Respiratory and Nervous System Science, Biomedical Laboratory Science, Graduate School of Medical and Dental Sciences, Tokyo Medical and Dental University, Tokyo, Japan; c Department of Neurology, Gifu University Graduate School of Medicine, Gifu, Japan; d Department of Neurology and Neurobiology of Aging, Kanazawa University Graduate School of Medical Science, Kanazawa, Japan

**Keywords:** Electroencephalogram, focal spike-and-wave complexes, MM2 cortical form, periodic sharp wave complexes, sporadic Creutzfeldt-Jakob disease

## Abstract

Periodic sharp wave complexes (PSWCs), identified using electroencephalography, are observed in less than half of patients with the methionine homozygosity type 2 cortical (MM2c) form of sporadic Creutzfeldt-Jakob disease (sCJD), and only at a later stage of the disease. In this study, we identified early and specific markers on the electroencephalograms (EEGs) of patients with MM2c-sCJD. We retrospectively investigated the clinical records, EEGs, and magnetic resonance imaging (MRI) scans of patients diagnosed with sCJD and compared the EEG findings of MM2c-sCJD and MM1/classic sCJD groups. The records of six patients with MM2c-sCJD and eight with MM1/classic sCJD were included. The median ages of onset in the MM2c- and MM1/classic sCJD groups were 75.0 (range, 60–83) and 72.5 (range, 51–74) years, respectively, and the average durations between disease onset and the first EEG were 9.17 (range, 4–15) and 1.88 (range, 1–4) months, respectively. Focal sharp waves and/or focal spike-and-wave complexes in the brain regions corresponding with cortical hyperintensities on MRI scans were identified on the EEGs of patients with MM2c-sCJD in the early stages of disease progression. In contrast, EEGs of patients in the early stages of MM1/classic sCJD showed lateralized or generalized diffuse sharp waves and spike-and-wave complexes, which were not limited to cortical hyperintensities identified with MRI scans. Our findings indicate that focal sharp waves and/or focal spike-and-wave complexes on the EEGs of patients in the early phase of MM2c-sCJD are characteristic of the disease, suggesting the possible usefulness of this characteristic for early diagnosis.

## Introduction

Creutzfeldt-Jakob disease (CJD) is the most common form of prion disease or transmissible spongiform encephalopathy (TSE) [1]. It is a rare and fatal disease caused by the abnormal scrapie isoform of the prion protein (PrP^Sc^). Human prion diseases are classified into three subtypes: sporadic, genetic (familial), and acquired (infected). Sporadic CJD (sCJD) constitutes approximately 80% of all prion disease and is not associated with a mutation in the prion protein gene (*PRNP*) [2]. sCJD is classified into different types based on the polymorphism at codon 129 of the *PRNP* gene, as follows: methionine homozygosity (MM), valine homozygosity (VV), and methionine/valine heterozygosity (MV). These types are further classified into type 1 and type 2 based on the molecular weight of proteinase-K resistant pathologic prion protein (PrP^Sc^). Therefore, sCJD can be classified into six types: MM1, MV1, VV1, MM2, MV2 and VV2 [3].

Approximately 70% of sCJD patients have the MM1 or MV1 type, which is also called classic CJD. MM2-type sCJD (MM2-sCJD) presents with at least two further pathologic phenotypes: MM2-cortical (MM2c) and MM2-thalamic (MM2th) forms [4]. MM2c-sCJD presents as relatively slowly progressing dementia, without visual or cerebellar signs of impairment. Most patients with MM2c-sCJD do not meet the existing clinical criteria for the diagnosis of sCJD [5]; thus, distinct clinical criteria have been proposed [4]. Magnetic resonance imaging (MRI), cerebrospinal fluid (CSF) analysis, and electroencephalogram (EEG) have been reported as useful methods for the diagnosis of MM2c [6].

MM2c-sCJD is characterized by cortical ribbon hyperintensity in diffusion-weighted imaging (DWI) and fluid-attenuated inversion recovery (FLAIR) MRI of the cerebral cortex. This pattern is not usually seen in the basal ganglia [4,6]. The total tau and 14-3-3 protein levels in the CSF were reportedly elevated in approximately 90% of patients [7–9], and periodic sharp wave complexes (PSWCs) on the EEG were observed in 42% of patients with MM2c-sCJD throughout the disease course, including later stages of the disease [6]. Although some of these characteristics have been proposed as criteria for the diagnosis of MM2c-sCJD [4], not all patients with MM2c-sCJD necessarily present with these characteristics, especially in the early stages of the disease. Most notably, PSWCs on EEGs were not observed in the early stages [6]. Therefore, the aim of this study was to find specific EEG signatures at an early stage in patients with MM2c-sCJD.

## Results

Six patients with MM2c-sCJD and eight with MM1/classic sCJD were included in the study. One patient who had been clinically diagnosed with the Heidenhain variant of MM1-sCJD was pathologically diagnosed with MM1 + 2c-sCJD upon autopsy. This patient was excluded from the comparative analysis of this study. All patients were confirmed by genetic analysis to have homozygosity for methionine at codon 129 and no prion protein gene mutation. Two patients in the MM2c-sCJD group, one of whom we previously reported [10], and three patients in the MM1-sCJD group, underwent autopsy.

To confirm the type of PrP^res^, brain homogenates were analysed by Western blotting using the 3F4 antibody as well as type 1 and type 2 PrP^Sc^-specific antibodies [10]. Histopathological analyses of the brains of patients with MM2c-sCJD revealed spongiform changes comprised of large confluent vacuoles, and perivascular- and rough plaque-type PrP^Sc^ deposition in the cerebral cortices with preserved inferior olivary nuclei [10]. Spongiform changes with small vacuoles and synaptic-type PrP^Sc^ deposition in the cerebral cortices were identified in the brains of patients with MM1-sCJD. Spongiform changes comprised of predominantly small vacuoles with synaptic-type PrP^Sc^ deposition and some amount of large confluent vacuoles with perivacuolar PrP^Sc^ deposition were identified in the brain of the patient with MM1 + 2c-sCJD.

The median age of patients at onset in the MM2c-sCJD (75.0 years; range, 60–83) and MM1/classic sCJD groups (72.5 years; range, 51–74) were not significantly different (*p* = 0.17). However, the average periods between disease onset and the first EEG in the MM2c-sCJD (9.17 months; range, 4–15) and MM1/classic sCJD groups (1.88 months; range, 1–4) were significantly different (*p* = 0.003), indicating that disease progression in patients with MM2c-sCJD was relatively slow, thus delaying their first visit to a hospital. The cognitive decline of each patient was evaluated using the Mini-Mental State Examination or the revised Hasegawa dementia rating scale [11]. Cognitive tests, which were performed approximately 10 months later for patients in the MM2c-sCJD group than those in the MM1/classic sCJD group, indicated an apparent cognitive decline in both groups (Table 1). The clinical features are summarized in Table 1.

**Table 1.** Clinical features of MM2 c and MM1/classic sCJD groups.

		MM2 c (N = 6)	MM1/classic CJD (N = 8)	*P*
Male		4/6 (66.7%)	2/8 (25%)	
Age at onset (median, range)		75 (60–83)	72.5 (51–74)	0.17
Time of first consultation from disease onset (months; mean, range)		8.5 (4–17)	1.75 (1–3)	0.002
Time of first EEG from disease onset (months; mean, range)		9.17 (4–15)	1.88 (1–4)	0.003
Diagnosis				
Definite		2/6 (33.30%)	3/8 (37.5%)	
Probable		3/6 (50%)	5/8 (62.5%)	
Possible		1/6 (16.7%)	0/8 (0%)	
Clinical symptoms and signs**				
Cognitive function (mean, range)***	(MMSE)	9.25 (0–23)	9 (0–20)	
	(HDS-R)****	4.5 (0–12)	10 (0–26)	
Progressive dementia	(Rapid)	1/6 (16.7%)	8/8 (100%)	
	(Slow)	5/6 (83.3%)	0/8 (0%)	
Myoclonus		1/6 (16.7%)	8/8 (100%)	
Visual or Cerebellar signs		5/6 (83.3%)	7/8 (87.5%)	
Pyramidal/extrapyramidal signs		2/5 (40%)	5/8 (62.5%)	
Akinetic mutism		0/6 (0%)	0/8 (0%)	

**Clinical signs and symptoms, except cognitive function, were observed one year and 1 to 3 months after disease onset in the MM2c-sCJD and the MM1/classic CJD groups, respectively.

***Cognitive function was examined between 4 to 17 months and between 1 to 3 months after disease onset, in the MM2c-sCJD and the MM1/classic CJD groups, respectively.

****Imai Y, Hasegawa K. The revised Hasegawa’s dementia scale (HDS-R)-evaluation of its usefulness as a screening test for dementia. J Hong Kong Coll Psychiatr.1994;4 (2):20–24.

EEG: electroencephalogram, sCJD: sporadic Creutzfeldt-Jakob disease, MM2c: methionine homozygosity type 2 cortical form, MMSE: Mini-Mental State Examination.

**Figure 1.**
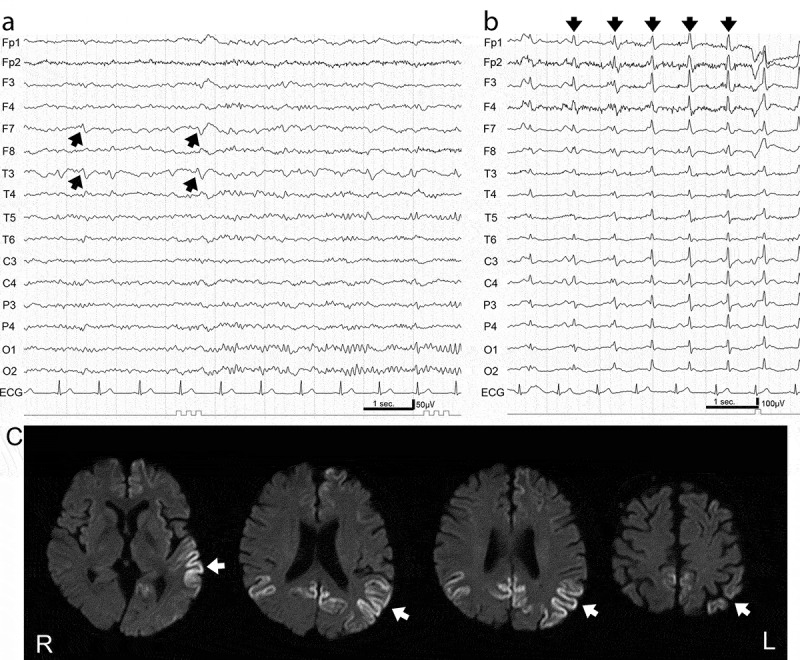
Electroencephalogram (EEG) and magnetic resonance imaging (MRI) in MM2c-sporadic Creutzfeldt-Jakob disease.

**Figure 2.**
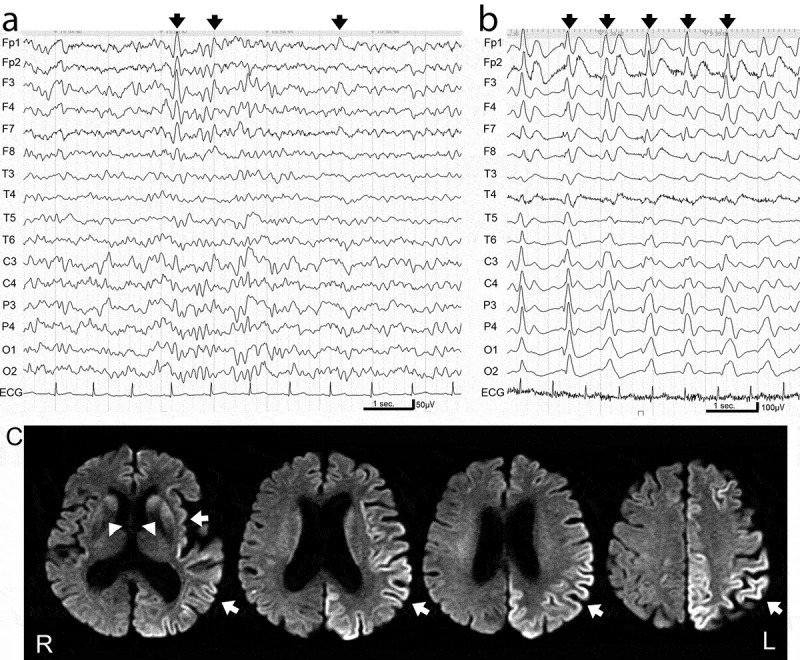
Electroencephalogram (EEG) and magnetic resonance imaging (MRI) in MM1-sporadic Creutzfeldt-Jakob disease.

Focal sharp waves and/or focal spike-and-wave complexes were identified on EEGs corresponding to brain regions showing cortical hyperintensity in MRI scans during early stages (4–15 months after disease onset) in patients in the MM2c-sCJD group. Cognitive impairment was also detected in these patients (Figure 1(a,c), Supplementary Table 1). In contrast, lateralized sharp or triphasic waves and spike-and-wave complexes, probably due to variations in pathological progression, were observed in EEGs during early stages (1–2 months after onset) in patients in the MM1/classic sCJD group (Figure 2(a)). These paroxysmal waves were not associated with cortical hyperintensities in brain MRI scans and were observed before the emergence of the PSWCs. In our study, three patients (50%) in the MM2c-sCJD group had PSWCs (as seen with EEG) only in the later stages (27–58 months after onset), whereas seven patients (87.5%) in the MM1/classic sCJD group showed PSWCs from the very early stages (1–4 months after onset). None of the patients with MM2c-sCJD in the early phase of the study exhibited convulsions; however, most of the patients in the study, both with MM1/classic-sCJD and MM2c-sCJD, showed myoclonus in their extremities coincident with the emergence of PSWCs on EEG.

(a) EEG readings in the early stage (10 months after disease onset) showed background activity of 10–12 Hz and 75–100 μV and focal discharges (arrows) at F7 and T3, corresponding to hyperintense lesions detected by MRI. (b) EEG readings in the late stage (58 months after disease onset) showed low amplitude background activity at 9–10 Hz and periodic sharp wave complexes at a frequency of 1 Hz (arrows). (c) Diffusion-weighted MRI in the early stage (7 months after disease onset) showed multiple focal hyperintense signals (left-dominant temporo-occipital and bilateral medial occipital cortices in this case; arrows).

(a) EEG readings in the early stage (1 month after disease onset) showed background activity of 6–8 Hz and 50–75 μV and lateralized frontal dominant sharp or triphasic waves (arrows). (b) EEG readings in the later stage (4 months after disease onset) showed poorly organized background activity and periodic sharp wave complexes at a frequency of 1 Hz (arrows). (c) Diffusion-weighted MRI in the early stage (2 months after disease onset) showed hyperintense signals in the cortices and basal ganglia (arrows).

Slow background activity was observed in the first EEG recording in four patients (66.7%) in the MM2c-sCJD group and in six patients (75%) in the MM1/classic sCJD group. All patients in the MM2c-sCJD group showed focal cortical ribbon signs in the cerebral cortex without hyperintensity of the basal ganglia or thalamus. The patient with MM1 + 2c showed background α to θ wave activity with sharp and wave complexes, in the posterior temporal region (as seen with EEG) 1 month after disease onset; further, occipital dominant periodic diffuse synchronous high amplitude sharp and wave complexes were seen via EEG 2 months after disease onset. Elevation of the total tau protein level in the CSF was detected in one patient (16.7%) in the MM2c-sCJD group and in eight patients (100%) in the MM1/classic sCJD group. CSF tests were positive for 14-3-3 proteins in three patients (50%) in the MM2c-sCJD group and for eight patients (100%) in the MM1/classic sCJD group. The laboratory and neuroimaging findings of each patient are listed in Supplementary Table 2.

## Discussion

Eleven of the 14 patients included in our study showed epileptic discharges at the time of the first EEG. PSWCs typically observed in sCJD are symmetrical and generalized triphasic, biphasic, or mixed complexes, occurring about every second on the EEG. Detection of PSWCs in EEG recordings is associated with CJD with a sensitivity of 67% and specificity of 86% [12]. Lateralized PSWCs, frontal intermittent rhythmical delta activity, and non-specific diffuse slowing background have been described as abnormalities that are commonly found in the early stages of MM1/classic sCJD [13–15].

One patient with the Heidenhain variant of sCJD was reported to show periodic complexes on EEG at the occipital regions during the six-week period following disease onset [16]; however, only the independent component analysis technique was capable of detecting periodic and epileptiform discharges in the early stage of sCJD [17]. A few case reports describing patients with CJD and non-convulsive status epilepticus have been published, showing continuous diffuse spikes, rhythmic sharp waves and sharp-and-slow wave complexes [18–20]; in one case, a patient was administered diazepam and EEG abnormalities were completely abolished. In contrast, to the best of our knowledge, only few case reports describing specific EEG findings of patients with MM2c-sCJD have been published. In particular, the case of only one patient with MM2c-sJCD who presented with intermittent rhythmic generalized slowing (as seen with long-term EEG monitoring) has been described [21]. Lateralized PSWCs are detected in the early stage of the disease for patients with MM1/classic sCJD (Figure 2(a)), while the characteristic PSWCs, described above, are observed approximately 2–3 months after disease onset (Figure 2(b)).

Occasionally, epileptic paroxysmal patterns on EEGs, such as sharp waves and spike-and-wave discharges, have been reported in patients in the early stage of sCJD [19], but there is no correlation between the hyperintense regions on MRI scans and the foci of discharges on EEGs. Focal sharp wave and focal spike-and-wave complexes do not seem to be associated with physical convulsion in the early phase of the disease for patients with MM2c-sCJD; PSWCs detected in patients with either type of sCJD were probably associated with myoclonus. Interestingly, EEG findings of the patient with MM1 + 2c-sCJD were similar to that of patients with MM2c-sCJD in the first month after onset; however, EEG in the second month revealed 1 Hz periodic synchronous paroxysm in this patient that resembled those of patients with MM1-sCJD. The clinical duration of this patient was only 3 months, which was consistent with MM1-sCJD. EEG findings of patients with MM1 + 2c-sCJD have previously been reported to show PSWCs in 53% of cases [22]. It is probable that clinical and pathological features of this mixed form are defined by the component ratio of MM1- and MM2-type prion proteins.

The clinical presentation of MM2c-sCJD is characterized by a relatively slow progression, cortical hyperintensity on diffusion-weighted MRI without hyperintensity of the basal ganglia, and elevated 14-3-3 protein levels in the CSF [4]. In our study, the average duration between disease onset and the first EEG in patients with MM2c-sCJD was 9.17 months, which was significantly longer than that of patients with classic/MM1 sCJD (1.88 months). Histological samples of patients with MM2c-sCJD showed spongiform changes characterized by large and confluent vacuoles, gliosis, and necrosis, predominantly observed in the cerebral cortex [23]. A study comparing autopsy reports of seven patients with MM2c-sCJD found no correlation between disease duration, grade and pattern of histological phenotype, and abnormalities in MRI scans. However, the indications of histological examinations might not have been reflected in the pathological condition when the MRI scans were performed. This is because the MRI scans, the results of which are documented in the report, were performed 5.5 months before the deaths of all seven patients [6]. Notably, histological examination in one of the autopsied cases of MM2c-sCJD with a disease duration of 6 months revealed that the extent of spongiform and neurodegenerative changes was more severe in the posterior cortex, whereas DWI had shown hyperintensity in the anterior cortex [23].

Slow-progressing pathophysiology caused by type 2 PrP^Sc^ is associated with slow clinical progression in patients with MM2c-sCJD. However, hyperintensities observed in DWI of MRI could represent relatively fast-growing lesions in the brain. These lesions probably caused long-term focal stimulation of the neuronal network in the patients’ brains and resulted in subsequent focal epileptic activity. Notably, higher amplitude laterality of PSWCs was observed on the EEGs of some patients with MM1/classic sCJD at stronger hyperintensity lesions on DWI, which is associated with more severe spongiform changes with gliosis, neural loss in the cortex [24].

Early diagnosis of MM2c-sCJD is considered difficult because existing diagnostic criteria for CJD are not useful in cases of MM2-sCJD. CSF tests during the early stages may not show increased levels of 14-3-3 and tau proteins [25]. In the present study, the first CSF test that was performed 10 months after disease onset was negative for increased levels of 14-3-3 protein in one patient. However, the third CSF test that was performed 28 months after disease onset was positive. In addition, PSWCs did not appear on the EEGs of any of the six patients with MM2c-sCJD in the early stages, and only three patients showed PSWCs after more than one year after disease onset (Table 2). Focal discharges on EEG are highly characteristic findings and could be useful as an early diagnostic marker of MM2c-sCJD. With regard to the differential diagnosis from a few conditions that mimic sCJD, such as Hashimoto’s encephalitis [26] and status epileptics [27], that sometimes show focal hyperintensities on DWI and focal discharges on EEG resembling those of MM2c-sCJD, a combined evaluation consisting of apparent diffusion coefficient mapping or arterial spin labelling of MRI with DWI and EEG is useful for diagnosis [28,29].

**Table 2.** Laboratory and neuroimaging findings of MM2 c and MM1/classic CJD groups.

		MM2 c (N = 6)	MM1/classic CJD (N = 8)
First EEG			
slowing		4/6 (66.7%)	6/8 (75%)
paroxysm	(Focal sharp waves or focal spike and wave complexes)	6/6 (100%)	0/8 (0%)
	(Lateralized diffuse sharp waves or lateralized diffuse spike and wave complexes)	0/6 (0%)	5/8 (62.5%)
	(PSWCs)	0/6 (0%)	3/8 (37.5%)
Follow up EEG			
slowing		6/6 (100%)	8/8 (100%)
paroxysm	(Focal sharp waves or focal spike and wave complexes)	3/6 (50%)	0/8 (0%)
	(Lateralized diffuse sharp waves or lateralized diffuse spike and wave complexes)	0/6 (0%)	1/8 (12.5%)
	(PSWCs)	3/6 (50%)	7/8 (87.5%)
Abnormal signal on MRI			
CO		6/6 (100%)	8/8 (100%)
BG		0/6 (0%)	5/8 (62.5%)
TH		0/6 (0%)	0/8 (0%)
Cerebrospinal fluid			
T-tau protein positivity		1/6 (16.7%)	8/8 (100%)
14-3-3 protein positivity		3/6 (50%)	8/8 (100%)
RT-QuIC positivity		4/5 (80%)	6/6 (100%)
Genetics			
Codon129	Met/Met	6/6 (100%)	5/5 (100%)
Codon219	Glu/Glu	6/6 (100%)	5/5 (100%)
Pathological anatomy		2/6 (33.3%)	3/8 (37.5%)

BG: basal ganglia, CO: cerebral cortex, EEG: electroencephalogram, Glu: glutamic acid, Met: methionine, MRI: magnetic resonance image, PSWC: paroxysmal sharp wave complex, RT-QuIC: real-time quaking-induced conversion, TH: thalamus, sCJD: sporadic Creutzfeldt-Jakob disease, MM2 c: methionine homozygosity type 2 cortical form.

We would like to suggest modifying the previously proposed MM2c-sCJD diagnostic criteria to the following [4]: (1) slow-progressing dementia, (2) methionine homozygosity at codon 129 of the *PRNP* gene without mutations, (3) DWI showing hyperintensities confined to the cortex of the brain, (4) delayed onset, (5) focal discharges on EEG, and (6) no more than two out of the following four clinical features within 6 months after onset – (a) myoclonus, (b) pyramidal or extrapyramidal sign, (c) cerebellar ataxia or visual impairment, and (d) akinetic mutism.

The present study has a few limitations. First, the sample size was small due to the low prevalence of MM2c-sCJD; therefore, we could not analyse the pathological relevance of the periodic waves on the EEGs of patients with MM2c-sCJD. Second, the interval between disease onset and a patient’s first EEG was not consistent amongst patients because this was a retrospective multicenter observational study. A prospective study with a larger cohort will be required to draw more robust conclusions.

To conclude, we report that focal sharp waves and/or focal spike-and-wave complexes on EEGs were detected in all patients with MM2c-sCJD, corresponding to the cortical hyperintense regions detected by MRI in early stages of clinical course. Three patients (50%) had PSWCs in the later stages; hence, focal sharp waves and/or focal spike-and-wave complexes could be a characteristic and useful diagnostic marker for early stages of the disease. We acknowledge, however, that the sample size in our study was small. Furthermore, only two patients were pathologically confirmed with MM2c-sCJD; therefore, a more detailed study with a larger sample size is warranted.

## Patients and methods

The clinical records, MRI scans, and EEGs of patients admitted to Tokyo Medical and Dental University Hospital, Gifu University Hospital, and Kanazawa University Hospital who were diagnosed with sporadic CJD between June 2007 and February 2020 were retrospectively investigated. Patients were classified into MM1/classic sCJD and MM2c-sCJD groups according to their clinical presentation, laboratory findings, radiological findings, neuropathology, and genetic analysis. Patients were classified as having the MM1/classical form of sCJD based on the World Health Organization criteria [30] or via pathological analysis, and MM2c-sCJD was diagnosed based on pathological analyses or the clinical criteria proposed by Hamaguchi et al. [4]. None of these patients had a family history of prion disease or a history of neurosurgery early (non-specific symptomatic stage), middle (symptomatic stage), and late (terminal stage), according to their clinical features. The protein levels of 14-3-3 and tau in the CSF were measured using previously described methods [31]. Analyses of *PRNP* and neuropathology were performed as described previously [2,32].

EEG findings of the MM2c-sCJD group and those of the MM1/classic sCJD group were compared. EEGs had been performed at least twice for each patient using the 10–20 electrode placement system. Brainwaves were classified according to the criteria described by Teplan [33]. The Mann–Whitney U and Fisher’s exact tests were used to compare the data between groups. All analyses were performed using R version 2.8.1 (R Foundation for Statistical Computing). P-values (*P*) <0.05 were considered to be statistically significant.

The family of each patient gave informed consent for patient inclusion in the study. The study protocol was approved by the Institutional Ethics Committee of Tokyo Medical and Dental University and was performed in accordance with the ethical standards of the 1975 Declaration of Helsinki.

## Supplementary Material

Supplemental Material
