# Supplementary material for: Focal sharp waves are a specific early-stage marker of the MM2-cortical form of sporadic Creutzfeldt-Jakob disease
Source: Prion. 2020 Aug 13;14(1):207–13. doi: 10.1080/19336896.2020.1803516 (PMC7518755; doi:10.1080/19336896.2020.1803516)
Supplement: Supplemental Material [file KPRN_A_1803516_SM7434.docx]

| **Supplementary Table 1. Early clinical features in 14 patients** | | | | | | | | | | | | | |
| --- | --- | --- | --- | --- | --- | --- | --- | --- | --- | --- | --- | --- | --- |
| Pt no. | Diagnosis | Grades of diagnosis | Sex | Onset, y | First consultation, mo* | Clinical symptoms and signs** | | | | | | |  |
|  |  |  |  |  |  | Cognitive function*** | | Progressive dementia | Myoclonus | Visual or  Cerebellar signs | Pyramidal/  extrapyramidal signs | Akinetic mutism |  |
|  |  |  |  |  |  | MMSE | HDS-R |  |  |  |  |  |  |
| 1 | MM2c | Probable | M | 69 | 14 | impossible | | slow | － | ＋ | ＋ | － |  |
| 2 | MM2c | Probable | M | 60 | 7 | n.a | | slow | － | － | － | － |  |
| 3 | MM2c | Probable | F | 75 | 5 | n.a | 6 | slow | － | + | － | － |  |
| 4 | MM2c | Probable | M | 83 | 4 | 14 | 12 | rapid | － | ＋ | ＋ | － |  |
| 5 | MM2c | Definite | M | 83 | 17 | impossible | | slow | ＋ | ＋ | n.a | － |  |
| 6 | MM2c | Definite | F | 75 | 4 | 23 | n.a | slow | － | + | － | － |  |
| 7 | MM1 | Definite | F | 66 | 1 | n.a | 26 | rapid | ＋ | ＋ | ＋ | － |  |
| 8 | MM1 | Definite | F | 73 | 2 | n.a | 1 | rapid | + | + | － | － |  |
| 9 | MM1 | Definite | M | 77 | 3 | impossible | | rapid | + | + | + | － |  |
| 10 | Classic sCJD | Probable | F | 74 | 1 | impossible | | rapid | + | + | － | － |  |
| 11 | Classic sCJD | Probable | M | 72 | 1 | n.a | 22 | rapid | + | + | － | － |  |
| 12 | Classic sCJD | Probable | F | 74 | 1 | n.a | 3 | rapid | ＋ | － | ＋ | － |  |
| 13 | Classic sCJD | Probable | F | 55 | 3 | 20 | 13 | rapid | ＋ | ＋ | ＋ | － |  |
| 14 | Classic sCJD | Probable | F | 51 | 2 | 16 | 15 | rapid | ＋ | ＋ | ＋ | － |  |
| sCJD: sporadic Creutzfeldt-Jakob disease, MM2c: methionine homozygosity type 2 cortical form.  *Time from disease onset.  **Clinical signs and symptoms, except cognitive function, were examined at 1 year and 1 to 3 months after disease onset in the MM2c-sCJD group and the MM1/classic CJD group, respectively.  ***Cognitive function was examined 4 to 17 months and 1 to 3 months after disease onset in the MM2c-sCJD group and the MM1/classic CJD group, respectively.  HDS-R: revised Hasegawa's dementia scale, MMSE, Mini-Mental State Examination. | | | | | | | | | | | | | |

| **Supplementary Table 2. Laboratory and neuroimaging findings in 14 patients** | | | | | | | | | | | | | | |
| --- | --- | --- | --- | --- | --- | --- | --- | --- | --- | --- | --- | --- | --- | --- |
| EEG | | | Abnormal signal on MRI | | | | Cerebrospinal fluid | | | | Genetics | | Pathological anatomy |  |
| slowing | paroxysm | DD, mo | CO | BG | TH | DD, mo | T-tau (pg/ml) | 14-3-3 (µg/ml) | RT-QuIC | DD, mo | Codon129 | Codon219 |  |  |
| ＋ | Focal sharp waves | 14 | ＋ | － | － | 14 | － (628) | － | ＋ | 10 | Met/Met | Glu/Glu | － |  |
|  |  |  |  |  |  |  | － (610) | － |  | 14 |  |  |  |  |
| ＋ | PSWCs | 27 |  |  |  |  | － (908) | ＋ (1518.1) |  | 28 |  |  |  |  |
| － | Focal Spike and wave complexes | 10 | ＋ | － | － | 7 | － (1014) | ＋ (1035) | ＋ | 7 | Met/Met | Glu/Glu | － |  |
| ＋ | PSWCs | 58 |  |  |  |  |  |  |  |  |  |  |  |  |
| ＋ | Focal sharp waves,  Focal spike and wave complexes | 10 | ＋ | － | － | 7 | － | － | ＋ | 9 | Met/Met | Glu/Glu | － |  |
| ＋ | PSWCs | 36 |  |  |  |  |  |  |  |  |  |  |  |  |
| ＋ | Focal sharp waves | 4 | ＋ | － | － | 12 | － (728) | － (<500) | － | 1 | Met/Met | Glu/Glu | － |  |
| ＋ | Focal sharp waves | 13 |  |  |  |  |  |  |  |  |  |  |  |  |
| ＋ | Focal sharp waves | 15 | ＋ | － | － | 14 | ＋ (>2200) | ＋ (>500) | ＋ | 15 | Met/Met | Glu/Glu | ＋ |  |
| ＋ | Focal sharp waves | 17 |  |  |  |  |  |  |  |  |  |  |  |  |
| － | Focal spike and wave complexes | 4 | + | － | － | 4 | － (960) | － | n.a | 4 | Met/Met | Glu/Glu | + |  |
| + | Focal sharp waves | 35 |  |  |  |  |  |  |  |  |  |  |  |  |
| － | Lateralized diffuse sharp waves | 1 | ＋ | ＋ | － | 1 | ＋ (>2200) | ＋ (1146.4) | ＋ | 1 | Met/Met | Glu/Glu | ＋ |  |
| ＋ | PSWCs | 4 |  |  |  |  |  |  |  |  |  |  |  |  |
| ＋ | Lateralized diffuse spike and wave complexes | 2 | ＋ | － | － | 2 | ＋ (3180) | ＋ (676) | n.a | 2 | Met/Met | Glu/Glu | ＋ |  |
| ＋ | PSWCs | 3 |  |  |  |  |  |  |  |  |  |  |  |  |
| － | Lateralized PSWCs | 2 | + | － | － | 2 | ＋ (>2200) | ＋ (>500) | + | 4 | Met/Met | Glu/Glu | ＋ |  |
| + | PSWCs | 4 |  |  |  |  |  |  |  |  |  |  |  |  |
| + | PSWCs | 5 |  |  |  |  |  |  |  |  |  |  |  |  |
| ＋ | Lateralized diffusesharp waves | 1 | ＋ | － | － | 1 | ＋ (>2200) | ＋ (>500) | ＋ | 1 | Met/Met | Glu/Glu | － |  |
| ＋ | PSWCs | 2 |  |  |  |  |  |  |  |  |  |  |  |  |
| ＋ | PSWCs | 2 | ＋ | ＋ | － | 1.5 | ＋ (>2200) | ＋ (659.4) | ＋ | 1.5 | Met/Met | Glu/Glu | － |  |
| ＋ | PSWCs | 2 |  |  |  |  |  |  |  |  |  |  |  |  |
| ＋ | PSWCs | 1 | ＋ | ＋ | － | 2 | ＋ (>1300) | ＋ (7200.6) | n.a | 1 | n.a | | － |  |
| ＋ | PSWCs | 2 |  |  |  |  |  |  |  |  |  |  |  |  |
| － | Lateralized diffusesharp waves | 2 | ＋ | ＋ | － | 2 | ＋ (>2200) | ＋ (>500) | ＋ | 3 | n.a | | － |  |
| ＋ | PSWCs | 4 |  |  |  |  |  |  |  |  |  |  |  |  |
| ＋ | Lateralized diffuse sharp waves | 2 | ＋ | ＋ | － | 2 | ＋ (>2200) | ＋ (>500) | ＋ | 2 | n.a | | － |  |
| ＋ | Lateralized diffusesharp waves | 2 |  |  |  |  |  |  |  |  |  |  |  |  |
| BG: basal ganglia, CO: cerebral cortex, DD: disease duration at the time when each examination was performed, EEG: electroencephalogram, Glu: glutamic acid, Met: methionine, mo: months, MRI: magnetic resonance image, PSWC: paroxysmal sharp wave complex, RT-QuIC: real-time quaking-induced conversion, TH: thalamus. | | | | | | | | | | | | | | |
